# Overlapping volumes in re-irradiation for head and neck cancer – an important factor for patient selection

**DOI:** 10.1186/s13014-020-01587-3

**Published:** 2020-06-08

**Authors:** Anna Embring, Eva Onjukka, Claes Mercke, Ingmar Lax, Anders Berglund, Sara Bornedal, Berit Wennberg, Signe Friesland

**Affiliations:** 1 Department of Oncology, Karolinska University Hospital, Anna Steckséns gata 41, 171 76 Stockholm, Solna Sweden; 2 Department of Oncology-Pathology, Karolinska Institute, Stockholm, Sweden; 3 Medical Radiation Physics and Nuclear Medicine, Karolinska University Hospital, Stockholm, Sweden; 4 Epistat Epidemiology and Statistics Consulting, Uppsala, Sweden

**Keywords:** Re-irradiation, Head and neck cancer, Re-irradiation volume, Cumulative dose, Overlap, HNSCC

## Abstract

****Background**:**

There is a lack of consensus concerning the definition of re-irradiation and re-irradiation volumes in head and neck cancer (HNC). The aim of the present study is to introduce a more strict definition of the re-irradiated volume that might better predict the risk of serious side-effects from treatment.

****Methods**:**

Fifty-four consecutive patients re-irradiated for HNC cancer were retrospectively analysed. CT images were deformably registered and the dose distributions accumulated after conversion to EQD2. Patients with a cumulative dose of ≥100 Gy in the overlapping volume (V100) were included in the study. Survival data and radiation-related acute and late toxicities were recorded.

****Results**:**

The overall survival of all included patients at 2 and 5 years was 42.6 and 27.3% respectively and the progression free survival at 2 and 5 years was 32.5 and 28.5% respectively. The overall rate of any event of severe (grade ≥ 3) acute and late toxicity was 26 and 51%, respectively. We found that severe acute toxicity was more common in patients who had a larger overlapping volume (V100 > mean) where 43% of the patients experienced grade ≥ 3 acute toxicity, compared to the patients with smaller overlapping volumes (V100 < mean) where only 11% had severe toxicity (*p* = 0.02). The seemingly high rates of late toxicity in the present study could be due to the use of a more strict definition of re-irradiation. In previous studies also patients with low dose overlap are included and our results imply that there is a risk that previous studies might have overestimated the risk-benefit ratio in re-irradiation of HNC.

****Conclusions**:**

Our study describes the outcome of a patient material where a more strict definition of the re-irradiated volume is used. With this definition, which could better describe the volume of highest risk for serious complications, we found that larger such overlapping volumes result in an increase in severe acute side-effects. A clear definition of re-irradiation and re-irradiation volumes is of utmost importance for future studies of HNC to make results from different studies comparable.

## Background

Local recurrence is the predominant pattern of failure after treatment of advanced head and neck cancer (HNC) [1]. A recurrence often occurs in an area already treated with radiotherapy, which needs to be taken into account when considering different treatment options. The treatment method of choice is surgical resection [2], but for many patients, surgery is not possible due to unresectable tumour or co-morbidity. These patients may be offered palliative chemotherapy, associated with a median progression-free survival (PFS) of 3–4 months and a median overall survival (OS) of 6–10 months [3–5]. In recent years, immunotherapy has also emerged as a treatment option for patients with recurrent HNC. Immunotherapy can offer long-lasting remission for some patients, without the toxicity of chemotherapy, but only a limited group of patients with recurrent HNC benefit from immunotherapy and the knowledge regarding predictive factors in this group is still limited [6, 7].

Historically re-irradiation was avoided due to the risk of severe toxicity. However, several studies have now shown that re-irradiation for HNC is a treatment option for selected patients and can offer long-term disease control, or even cure [8–12]. In a multi-institution study including 412 patients with HNC, Ward et al. demonstrated an actuarial OS rate of 40% at 2 years [8], and in a recently published single-institution study by Rühle et al. the OS at 2 and 5 years was 52.3 and 34.3%, respectively, after re-irradiation for HNC [13]. However, re-irradiation for HNC still remains challenging mainly because of the concern of toxicity, and it would be useful to find predictive factors to guide patient selection. Deriving a consensus on patient selection from the literature is difficult due to varying or vague definitions of re-irradiation. Many studies suggest no definition of re-irradiation other than repeat radiotherapy in the head and neck area and it remains to be established what re-treatment parameters have clinical significance. It would be of great value if there was a consensus on how to define re-irradiation and re-irradiation volumes, as this would facilitate comparisons between different studies and in turn provide more solid data for patient selection. It is even possible that the value of re-irradiation in HNC has been overestimated due to the vague definition of re-irradiation in past studies, allowing the inclusion of patients who in fact had no overlapping volumes of high dose.

Intensity modulated radiotherapy (IMRT) has been shown to improve local control and survival in the treatment of head and neck cancer [14–17]. That more conformal techniques result in better patient outcome supports the hypothesis that the size of the irradiated volume might have an impact on patient outcome. It may be assumed that a relevant volume is the overlapping volume, i.e., the volume that is irradiated both at the initial treatment and then again at re-irradiation. This differs from the re-treatment volume, which is the volume treated at the time of re-irradiation, i.e., the planning target volume (PTV). However, as the latter is easier to obtain, this is the parameter typically studied in the literature, and it has been shown that the size of the PTV at re-treatment affects patient outcome [18, 19]. However, a large re-treatment volume might just represent a large tumour burden at re-irradiation and not necessarily a large overlapping volume. Today when treatment-planning data from the primary treatment most often is available electronically, it seems reasonable to move on from using the PTV at re-treatment as a surrogate for evaluating the re-irradiation volume. Therefore, the current study explores the impact on patient outcome from the overlapping volume rather than the re-treatment volume. Dosimetric data from both the primary treatment and the re-irradiation were gathered, making it possible to determine the overlapping volume.

In this work we propose a definition of the re-irradiated volume which better represents both treatments. The treatment outcome after re-irradiation for HNC in our institution, as per the proposed definition, is also evaluated in relation to patient- and treatment characteristics.

## Material and methods

### Patients

**Fig. 1:**
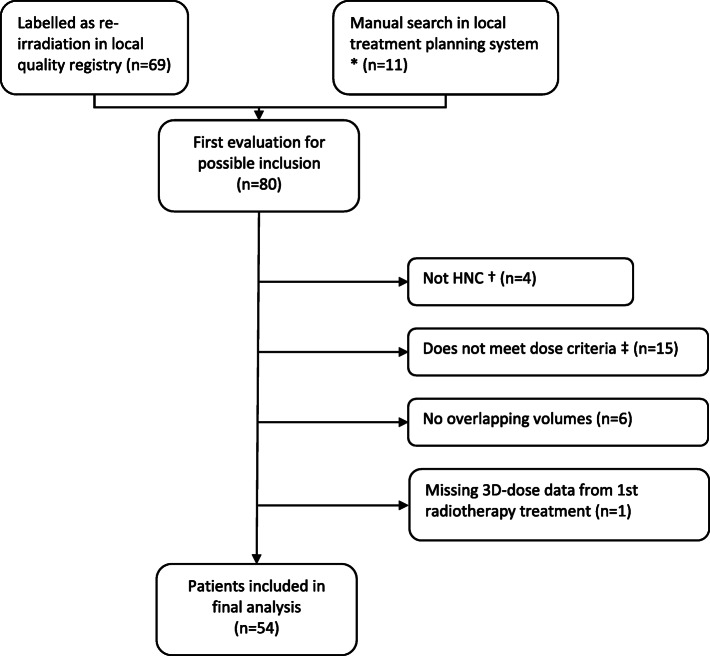
Flow chart of included patients. Abbreviations: HNC - Head and neck cancer. * All patients with a HNC diagnosis, who had at least 2 courses of radiotherapy and at least 1 course of radiotherapy with curative intent, were reviewed in the treatment planning system for inclusion, in order to find relevant patients who had not been recorded in the quality registry. † 1 malignant melanoma, 1 CNS tumour, 2 Merkel cell carcinoma (face). ‡ palliative radiotherapy

Fifty-four consecutive patients re-irradiated for HNC between 2011 and 2017 in our institution were retrospectively analysed. The inclusion criteria were initial radiotherapy with curative intent (≥60 Gy) for HNC, a second course of radiotherapy for recurrence of HNC or second primary HNC where the intent was to achieve cure or local control (≥40 Gy), and an overlapping volume of these two treatments (Fig. 1). Consistent with this definition of re-irradiation, the cumulative dose in what was considered the overlapping volume was ≥100 Gy in EQD2, i.e. V100. To account for different fractionation schedules and heterogeneous dose distributions, all radiotherapy doses are reported in an equivalent dose of 2 Gy fractions (EQD2) based on the linear-quadratic model [20], using α/β = 3 Gy.

A re-treatment dose ≥40 Gy was considered to reflect the clinician’s intent to achieve local control, in contrast to palliation; the same dose has been used in other studies of re-irradiation as a cut-off for significant re-irradiation dose [8, 18]. Thus, patients re-irradiated with palliative doses were excluded, as were patients re-irradiated for other tumours than HNC. One patient was also excluded because of missing data from the initial radiotherapy treatment. Three patients went on to have a second course of re-irradiation. Two of these patients had a second recurrence and one patient had a new second primary HNC. For these three patients the overlapping volumes and near-maximum doses were extracted from the total cumulative radiotherapy given. Patient and treatment characteristics at the initial treatment are reported in Table 1.

**Table 1:** Patient and treatment characteristics at first presentation. Tumour stage according to American Joint Committee on Cancer (AJCC) Cancer Staging Manual 7th edition

Patient and treatment characteristics at first presentation	Number	Percent (range)
**Male**	35	65
**Female**	19	35
**Median age (years)**	59	(33–81)
**Tumor site**		
Larynx	6	11
Oropharyngeal^a^	18	33
Nasopharyngeal	1	2
Hypopharyngeal	5	9
Oral cavity	17	31
Unknown primary	2	4
Sino/nasal	3	6
Salivary gland	2	4
**Histology**		
Squamous cell carcinoma	46	85
Adenoid cystic carcinoma	5	9
Other^b^	3	6
**Tumor Stage**		
I	5	9
II	11	20
III	7	13
IVa	28	52
IVb	3	6
**T-stage**		
0	2	4
1	9	17
2	22	41
3	4	7
4	17	31
**Median treatment dose (Gy)**	68	(60–79)

^a^Association with human papilloma virus (HPV): 11 HPV-positive, 2 HPV-negative, 5 HPV status unknown

^b^1 salivary duct cancer, 1 undifferentiated non-keratinizing cancer, 1 adenocarcinoma

In our institution approximately 230 patients with primary HNC are treated with curative intent every year. At first presentation patients are typically treated with chemoradiotherapy. Oropharyngeal cancer will typically receive definitive chemoradiotherapy without prior surgery and tumours of the oral cavity will typically undergo surgery before radiotherapy. During the studied period, patients with a tumour in the base of tongue often received a brachytherapy boost to the primary tumour after completing external beam radiotherapy.

The study was approved by the National Ethical Review Authority.

### Treatment

At re-irradiation, 93% of the patients were treated with highly conformal radiotherapy: either with IMRT or volumetric modulated arc therapy (VMAT), brachytherapy or a combination of external-beam radiotherapy and brachytherapy (Table 2). In the original treatments multiple techniques and modalities were used, including brachytherapy and external-beam therapy with 6 and 18 MV photon fields, and sometimes electrons. All treatment plans were computed tomography (CT) based and for all except four patients, both the original treatment plan and the re-treatment plan were available electronically. For the patients where the original treatment plan was not available electronically, plans were reconstructed on the re-treatment planning-CT from original treatment data available as print-outs (including field parameters, beam apertures, a selection of CT slices with isodoses and dose-volume histograms), to get complete dose data for all the included patients and all treatment courses. The reconstructions were performed by an experienced physicist; field shapes and weights were adapted to the anatomy in the available CT images, resulting in a plan reflecting the clinical practice at the time of treatment. Treatments were originally planned in Eclipse (Varian, USA), TMS (Helax, Sweden) or Pinnacle (Philips Radiation Oncology Systems, USA) and any reconstructions were made in Eclipse. All external-beam dose distributions were calculated in Eclipse using the AAA algorithm, also for plans imported from a different system. Brachytherapy treatments were planned in Oncentra (Elekta, Sweden).

**Table 2:** Patient and tumour characteristics at re-irradiation. Abbreviations: VMAT - Volumetric modulated arc therapy, IMRT - Intensity modulated radiotherapy, PTV – planning target volume

Patient and treatment characteristics at re-irradiation	Number	Percent (range)
**Median age at end of re-irradiation (years)**	63	(40–89)
**Median time between radiations (months)**	36	(5–177)
**Performance status**		
0	30	56
1	21	39
2	2	4
3	1	2
**Tumour**		
Local recurrence	37	69
Secondary primary tumour	17	31
**Surgery before re-irradiation**		
No	32	59
Primary tumour	11	20
Neck dissection	8	15
Primary tumour and neck dissection	3	6
**Systemic medical treatment**		
No	41	76
Induction chemotherapy	11	20
Concurrent chemotherapy (cisplatin)	1	2
Concurrent cetuximab	1	2
**Radiotherapy technique at re-irradiation**		
VMAT/IMRT	45	83
VMAT/IMRT + brachytherapy	3	6
3D conformal	4	7
3D conformal + brachytherapy	1	2
Brachytherapy	1	2
**Median re-irradiation dose (Gy)**	59	(40–71)
**Median cumulative near max dose, D1cc (Gy)**	129	(106–478)
**Median PTV at re-irradiation (cm**^**3**^**)**	145	(13–668)
**Median re-treated volume, V100 (cm**^**3**^**)**	90	(2–283)

The 3D-dose distributions of all included external-beam plans and brachytherapy plans were exported to an in-house application converting the dose in each voxel to EQD2. The CT-, structure- and dose data were imported into a research version of Raystation (Raysearch Laboratories, Sweden) where, for each patient, the planning-CT images were registered to the most recent CT used for external-beam planning, using a grey-level based non-rigid registration. The deformed dose-distribution from each treatment was calculated on the reference CT, finally giving the cumulative dose distribution in EQD2 including all the treatment plans. The dose to the hottest 1 cm^3^ (D1cc) of the patient volume, i.e. the near-maximum dose, as well as V100, were extracted from the cumulative dose distribution.

In this study, the accumulated dose from the original treatment and the re-treatment was derived by registering the planning-CT images non-rigidly and summing the deformed 3D dose. Non-rigid image registration has been shown to significantly improve the estimation of accumulated dose for re-irradiation [21]. While image registration in the head-and-neck region is challenging due to anatomical differences naturally appearing over time and due to different tilts of the head and different mouth fixation used for different treatment courses and modalities, non-rigid registration techniques appear to perform well for this anatomical site [22]. However, the CT images used for brachytherapy planning had extensive artefacts and a very limited field-of-view. For this reason, the brachytherapy planning-CT was never used as a reference image. Despite the challenges, visual evaluation of each non-rigid registration showed a good result.

### Oncologic and toxicity outcomes

Data of patient outcome were collected from a local quality registry, with prospectively gathered data, and supplemented with a review of medical records. Acute and late toxicities were graded according to Radiation Oncology Group (RTOG) and the European Organization for Research and Treatment of Cancer (EORTC) Radiation Morbidity Schema. Patients are invited for routine follow-up visits every 3 months the first 2 years after treatment, and then every 6 months for another 3 years. Patient outcome data were collected and recorded in the local quality registry every 6 months during this time. Toxicities were considered acute if presented within 90 days of the last day of re-irradiation. Any toxicities presenting later were considered late toxicities. Toxicities specifically investigated were mucositis, osteoradionecrosis, soft tissue necrosis, trismus, dysphagia and carotid blowout. The Eastern Cooperative Oncology Group (ECOG) Scale of Performance status (PS) was used to quantify the functional status of the patients.

Oncologic endpoints included OS and PFS. OS was defined as the time between the last day of radiotherapy to the time of death or the last date of clinical follow-up. PFS was defined as the time from the last day of re-irradiation to the time of progression, death or the last date of clinical follow-up. Progression was defined as either progression on diagnostic imaging, a positive biopsy or a clinical progression assessed by a clinician. Carotid blowout syndrome was defined as massive pharyngeal bleeding in the absence of local recurrence.

### Statistics

The Kaplan-Meier method was used to estimate OS and PFS from the last day of re-irradiation. Re-irradiation dose, overlapping re-treated volume, site of recurrence, PS at re-irradiation, size of PTV, interval between irradiations, definitive versus postoperative re-irradiation, recurrence versus second primary tumour and age were used as predictive variables. The chi-square test was used to test differences in toxicity. A test result below 5% was considered as statistically significant, and R version 3.6.1 was used for the data management and the analysis.

## Results

### Patient and treatment characteristics

A total of 54 patients were included in the analysis. Sixty-nine percent of the patients were treated for recurrent HNC and 31% for second primary HNC. The median time from first radiation treatment to re-irradiation was 36 months (range 5.2–177). The median follow-up time after re-irradiation was 20.1 months (range 0–69.9) in all patients and 54.1 months (range 34.3–66.3) in surviving patients. At closure of the database (December 20th, 2018) 11 patients were alive without disease and 2 patients were alive with disease. Causes of death are reported in Table 3.

**Table 3:** Cause of death

Cause of death	Number
Locoregional disease	27
Distant metastases	7
Other disease	4
Treatment complications	3
Total	41

**Fig. 2:**
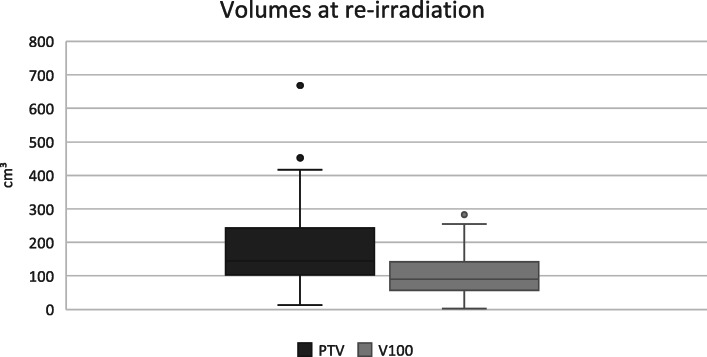
Volumes at re-irradiation. Lower and upper box boundaries indicate first and third quartile, respectively. The line inside the box marks median. Whiskers above and below the box indicate smallest and largest value within 1,5 times of the interquartile range from the quartiles. Points above whiskers indicates outliers outside of 1,5 times the interquartile range from the third quartile. Abbreviations: PTV – planning target volume, V100 – the volume at re-irradiation with a cumulative dose of ≥100 Gy (EQD2)

Median initial treatment dose was 68 Gy (range 60–79) and median re-irradiation dose was 59 Gy (range 40–71). For the external beam treatment, the median daily fractionation dose was 2 Gy at both primary treatment and re-irradiation. Nine patients had an additional brachy boost at the initial treatment and 5 patients had brachytherapy at re-irradiation. Median overlapping volume (V100) was 90 cm^3^ and median PTV at re-irradiation was 145 cm^3^ (Fig. 2). Patient and treatment characteristics are reported in Tables 1 and 2.

### Disease control and overall survival

**Fig. 3:**
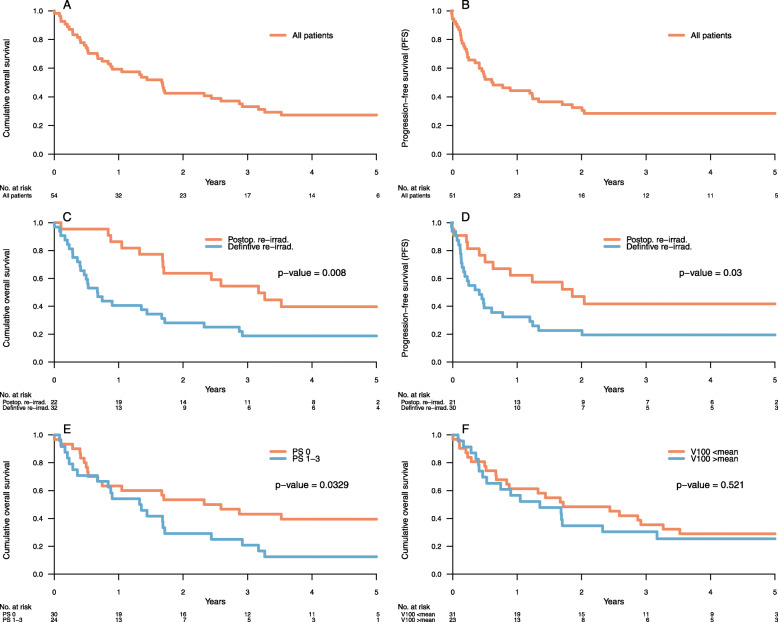
Kaplan-Meier curves of post re-irradiation overall survival (**a**) and progression free survival (**b**) for the entire cohort, and subgroups stratified on surgery (**b**, **c**) performance status (**d**) and size of the overlapping re-treated volume, V100 (**e**). Abbreviations: PS – performance status, V100 - the volume at re-irradiation with a cumulative dose of ≥100 Gy (EQD2), Abbreviations: Postop. – postoperative, re-irrad. – re-irradiation

The OS of all included patients at 2 and 5 years was 42.6 and 27.3% respectively and PFS at 2 and 5 years was 32.5 and 28.5% respectively. Three patients had progressive disease during treatment and were not eligible for PFS evaluation. Patients with a PS of 0 had a greater (*p* = 0.03) OS (53.3 and 39.5%, respectively, at 2 and 5 years) compared to patients with PS 1–3 (29.2 and 12.5%, respectively, at 2 and 5 years) (Fig. 3). Patients that were treated with postoperative re-irradiation had both better (*p* < 0.01) OS (63.6 and 39.7%, respectively, at 2 and 5 years) and better (*p* = 0.03) PFS (47.0 and 41.8%, respectively, at 2 and 5 years), compared to patients that were treated with definitive re-irradiation (OS at 28.1 and 18.8%, respectively, at 2 and 5 years and PFS at 22.6 and 19.4%, respectively, at 2 and 5 years) (Fig. 3). This study did not show a significant difference in OS or PFS related to the overlapping volume, PTV-size at re-irradiation, interval between irradiations, age at re-irradiation, D1cc or dose at re-irradiation (≥55 Gy vs < 55 Gy (EQD2 α/β = 10)).

### Toxicity

The overall rate of any event of severe (grade ≥ 3) acute and late toxicity was 26 and 51%, respectively. Three patients (5.6%) were thought to have died due to treatment-related toxicity. One patient died of acute radiation toxicity and 2 patients died of carotid blowout. The carotid blowouts presented 15 and 38 months after completion of re-irradiation. Severe acute toxicity was more common in patients who had a larger overlapping volume (V100 > mean) where 43% of the patients experienced grade ≥ 3 acute toxicity, compared to the patients with smaller overlapping volumes (V100 < mean) where only 11% had severe toxicity (*p* = 0.02) (Table 4). The majority of the cases of severe acute toxicities were mucositis (56%), followed by trismus (31%) and other (13%). We found no significant difference in severe acute toxicity related to the time interval between irradiations. No significant differences could be seen in late severe toxicity in correlation to the overlapping volume, nor could any significant differences be seen in the rate of acute or late severe toxicity in relation to D1cc or size of PTV at re-irradiation.

**Table 4:** Side effects after re-irradiation and the correlation to the overlapping volume. Abbreviations: V100 – the volume at re-irradiation with a cumulative dose of ≥100 Gy (EQD2). Toxicity was graded according to Radiation Oncology Group (RTOG) and the European Organization for Research and Treatment of Cancer (EORTC) Radiation Morbidity Schema

Side effects after re-irradiation^a^	Acute				Late			
	V100 < mean		V100 > mean		V100 < mean		V100 > mean	
	n	%	n	%	n	%	n	%
Grade 0–2	24	89	13	57	13	59	5	33
Grade ≥ 3	3	11	10	43	9	41	10	67

*p*-value = 0.023

*p*-value = 0.229

^a^ at least one grade 0–2, or grade ≥ 3 respectively

## Discussion

In our group of patients treated with re-irradiation for HNC the OS at 2 and 5 years was 42.6 and 27.3%, respectively. This is in accordance with other published data [8, 10], even though the definition of re-irradiation differs between studies. Our results show that patients with a good performance status (PS 0) have a significantly higher OS than patients with a worse performance status (PS 1–3). This is also in accordance with previously published literature. For example, in the study by Takiar et al., a multivariate analysis showed that PS > 1 was associated with unfavourable OS [23]. In our study, patients treated with re-irradiation postoperatively had a significantly better OS and PFS than patients treated with definitive re-irradiation. These results are also is in line with already published data [8, 24].

The rates of grade ≥ 3 toxicity were 26 and 51%, for acute and late toxicity respectively. The late side-effect rate is in the higher range of what is previously published. In a review article by Dionisi et al. including 3766 patients from 39 different re-irradiation studies in HNC, the pooled acute and late toxicity rates grade ≥ 3 were 32 and 29.3%, respectively [25]. One contributing factor to the high rates of late toxicity in the current study could be the relatively long follow-up. It is reasonable to assume that a longer follow-up time will detect more late toxicity and could be a reason for the lower toxicity rate in studies with shorter follow-up time [8, 18] compared to the current study. However, another contributing factor to the seemingly high rates of late toxicity could be our strict definition of re-irradiation. Patients with overlapping volumes in the low-dose area only are not included in our cohort, but only patients treated with ≥60 Gy at primary treatment and an overlapping dose of ≥40 Gy at re-irradiation. Previous studies have included also patients with overlapping volumes of lower doses. One could assume that these studies would detect less severe toxicity and lower rates of severe toxicity in the studied populations. Thus, our findings imply that late side effects in re-irradiated HNC patients is a greater problem than previous studies have suggested. Grade 5 toxicity was in level with other published data [10].

Several other groups have shown that re-irradiation is a treatment option for selected patients with recurrent or secondary primary HNC [8–10], but selecting patients for re-irradiation is challenging. Some prognostic factors have been identified: patients with a longer time interval between primary treatment and re-irradiation have better prognosis [24, 26], the absence of organ dysfunction (feeding tube or tracheostomy dependence) is favourable [8, 27], and higher doses at re-irradiation yield better outcomes [14, 24]. There are also various tools available to help guide clinicians in their decision making. Tanvetyanon et al. suggested a nomogram for predicting 24-month survival probability [27], and Ward et al. have constructed a nomogram for predicting severe late toxicity at two years after re-irradiation [28]. In the latter study it was suggested that late toxicity may be more dependent on patient- and disease factors than modifiable factors, since they found that dose, volume and fractionation had no significant impact on toxicity. However, with a more appropriate choice of volume parameter, based on the accumulated dose distribution, their conclusions may have been different; they only consider whether the patients have received elective node irradiation or not, rather than evaluating the actual overlapping volumes. In coherence with these results we find no correlation with the size of the PTV at re-irradiation and the toxicity rates. However, when investigating the actual re-treated volume, the overlapping volume, this was found to increase the rate of acute toxicity. This supports the hypothesis that the overlapping volume is a more appropriate volume to evaluate than the PTV in the re-irradiation setting. We are currently exploring the acute and late side effects in re-irradiation for HNC in more detail and investigate the relation to cumulative doses and different organs at risk. The results will be the subject of an accompanying publication.

Several studies have shown that the size of the target volumes has an impact on the outcome at re-irradiation. In a retrospective study by Takiar et al., 227 HNC patients treated with re-irradiation were reviewed and they found that a re-treatment volume (clinical target volume at re-irradiation) > 50 cm^3^ was associated with increased grade ≥ 3 toxicity [23]. In the study of Lee et al., based on 66 HNC patients re-irradiated with IMRT, smaller re-treatment volumes (PTV ≤100 cm^3^) had significantly reduced risk of severe late toxicity at 2 years (13% vs. 36%) [19]. Phan et al. conducted a study of re-irradiation with proton therapy of 60 HNC patients and this study showed that there was a significant association between a clinical target volume ≥ 50 cm^3^ and acute as well as late grade ≥ 3 toxicities [29]. A limitation of these studies is that they focus on the impact of target volumes at re-irradiation. In the current study, on the other hand, we hypothesized that a larger overlapping volume would have negative effect on patient outcome, such as OS and side effects, supported by the finding that IMRT is associated with a better OS compared to 3D-conformal radiotherapy [16, 17]. However, while in the current study a larger overlapping volume was associated with more acute toxicity it did not result in a lower OS or PFS. The failure to demonstrate such a relationship might be due to the moderate size of the dataset and/or the less specific nature of the latter endpoints. On the one hand, a large overlapping volume could lead to a higher risk of toxicity, but on the other hand, a large overlapping volume could also imply a large tumour volume, which in itself is associated with a poorer prognosis. In the former case, OS and PS might be limited due to the treatment while in the latter they are limited due to the disease. Another limitation of this study is its retrospective nature; despite careful scrutiny of the patient notes relevant information may not have been considered in the analysis. As previously referred to, there are also uncertainties in the accumulated dose distribution.

Nevertheless, this study and its definition of the overlapping re-treated volume could state an example on how re-irradiation and re-irradiation volumes should be defined in future studies. A clear definition on re-irradiation and re-irradiation volumes would make it easier to compare results and draw conclusions from different studies. Ultimately, this would make it easier to interpret the results from large studies and use the acquired knowledge in daily practice, when treating the individual patient. In our clinic we will calculate the overlapping volume for future HNC patients that are eligible for re-irradiation and consider this information valuable in selecting patients for re-irradiation with curative intent. This is important knowledge, because re-irradiation can mean cure for the correctly selected patient on one hand, but severe or even fatal toxicity for the poorly selected patient, on the other. The overlapping volume which can safely be treated is yet to be determined and should be the subject of future studies.

## Conclusions

The definition of re-irradiation and re-irradiation volumes is crucial in future studies in re-irradiation of HNC and a necessity to make results from different studies comparable. It is even possible that previous studies have overestimated the benefits of re-irradiation of HNC due to the vague definition of re-irradiation. Our study presents a definition of re-irradiation volume using the data and tools available in modern radiotherapy planning and suggests that larger overlapping volumes in re-irradiation of HNC results in an increase in severe acute side effects. The fact that we did not find a concomitant increase in late effects with increased overlapping volumes should be looked upon with caution and further evaluated with a longer follow up and in a larger patient material.

## Data Availability

Research data are stored in an institutional repository and will be shared upon reasonable request to the corresponding author.

## Abbreviations

HNC: head and neck cancer
V100: volume with a cumulative dose of ≥100 Gy
PFS: progression-free survival
OS: overall survival
IMRT: Intensity modulated radiotherapy
PTV: planning target volume
EQD2: equivalent dose of 2 Gy per fraction
VMAT: volumetric modulated arc therapy
CT: computed tomography
D1cc: dose corresponding to the hottest 1 cm^3^
RTOG: Radiation Oncology Group
EORTC: European Organization for Research and Treatment of Cancer
ECOG: Eastern Cooperative Oncology Group
PS: Performance status
AJCC: American Joint Committee on Cancer
